# Advancing Equity, Diversity, Inclusion, and Accessibility in a Patient-Oriented Kidney Research Network: A Can-SOLVE CKD Program Report

**DOI:** 10.1177/20543581261455663

**Published:** 2026-05-25

**Authors:** Keila Turino Miranda, Jocelyn Jones, Melanie D. Talson, James Scholey, Julie Wysocki, Meghan J Elliott, Letitia Pokiak, Maria Torrejon, Heather Harris, Selina Allu

**Affiliations:** 1 5620 Department of Kinesiology and Physical Education, McGill University, Montreal, Quebec, Canada; 2 Canadians Seeking Solutions and Innovations to Overcome Chronic Kidney Disease Bristish Columbia SUPPORT Unit, Vancouver, Bristish Columbia, Canada; 3 7938 Department of Physiology, Faculty of Medicine, University of Toronto, Toronto, Ontario, Canada; 4 103950 Kidney Foundation of Canada, Montreal, Quebec, Canada; 5 2129 Department of Medicine, O'Brien Institute of Public Health, Cumming School of Medicine, University of Calgary, Calgary, Alberta, Canada

**Keywords:** equity, diversity, inclusion, accessibility, kidney, patient-oriented research, audit, patient and public involvement

## Abstract

**Purpose of program:**

Despite increasing recognition that equity, diversity, inclusion, and accessibility (EDIA) principles are essential to research and improving health outcomes, there is little data to guide the incorporation of these principles in a research network. Here, we outline a collaborative approach taken by our kidney Strategic for Patient-Oriented Research (SPOR) network, Canadians Seeking Solutions and Innovations to Overcome Chronic Kidney Disease (Can-SOLVE CKD), to better understand the application of EDIA in a kidney research network.

**Sources of information:**

An internal network audit of EDIA strengths and future directions.

**Methods:**

Between June 2023 and January 2024, Can-SOLVE CKD’s EDIA working group completed a network-wide audit through seven virtual workshops, in which patient partners, research team members, and staff identified the network’s existing EDIA strengths and needs for improvement. Responses were collected on the Mural platform in real-time. Repeated or agreed-upon comments were displayed as multiple identical entries. A thematic analysis was conducted to extract key themes to inform the EDIA mobilization plan moving forward.

**Key findings:**

We collated feedback from 49 network members (16 patient partners, 19 research team members, and 14 staff) that included 441 individual responses from seven workshops. The thematic analyses revealed three key strengths: the inclusion and promotion of Indigenous voices and ways of knowing, patient partner participation empowerment and leadership, and network-wide acceptance of EDIA principles. Future direction themes included: increasing diversity of perspectives by outreach to under-represented communities, challenging biases against patient-oriented research, and improving accommodations for patient partner participation.

**Limitations:**

This analysis only captures the perspectives of people who are already within the network. While our network includes diverse people, some demographics and their respective perspectives may be missing.

**Implications:**

Many of Can-SOLVE CKD’s EDIA strengths can be attributed to a long-standing culture of meaningful inclusion of patient partners, strong leadership by Indigenous peoples and patient partners embedded in the network, as well as training, onboarding and compensation structures for patient partners. While current strategies have fostered a strong foundation, the next steps must ensure that inclusivity extends across all demographic populations and disciplinary boundaries. Participant suggestions, including grassroots outreach, hiring diverse leadership, and improving multilingual communication, demonstrate a willingness and readiness within the network to take actionable steps to further strengthen EDIA.

## Abstract (fr)

**Objectif du programme:**

Bien que les principes d’équité, de diversité, d’inclusion et d’accessibilité (EDIA) soient de plus en plus reconnus comme essentiels à la recherche et à l’amélioration des résultats de santé, les données guidant leur intégration au sein d’un réseau de recherche demeurent limitées. Nous décrivons ici une approche collaborative adoptée par notre réseau de recherche stratégique axée sur le patient (SPOR - Strategic for Patient-Oriented Research), le Can-SOLVE CKD (Canadians Seeking Solutions and Innovations to Overcome Chronic Kidney Disease), afin de mieux comprendre l’application des principes EDIA dans un réseau de recherche en néphrologie.

**Sources de l’information:**

Audit interne du réseau portant sur les forces existantes et les orientations futures en matière d'EDIA.

**Méthodologie:**

Entre juin 2023 et janvier 2024, le groupe de travail EDIA de Can-SOLVE CKD a réalisé un audit à l’échelle du réseau dans le cadre de sept ateliers virtuels, au cours desquels des patients partenaires, des membres de l’équipe de recherche et du personnel soignant ont identifié les forces et les besoins d’amélioration du réseau en matière d’EDIA. Les réponses ont été recueillies en temps réel sur la plateforme Mural. Les commentaires répétés ou consensuels apparaissaient sous forme d’entrées identiques multiples. Une analyse thématique a permis d’identifier les principaux thèmes pour guider le plan de mobilisation EDIA à venir.

**Principaux résultats:**

Les commentaires recueillis auprès de 49 membres du réseau (16 patients partenaires, 19 membres d’équipe de recherche, 14 membres du personnel) sont constitués de 441 réponses individuelles provenant de sept ateliers. Les analyses thématiques ont mis en évidence trois forces majeures: l’intégration et la promotion des perspectives et des savoirs autochtones, l’autonomisation et le leadership des patients partenaires, et l’adhésion aux principes d’EDIA à l’échelle du réseau. Les thèmes liés aux orientations futures comprenaient: la diversification des perspectives par l’engagement de communautés sous-représentées, la réduction des biais envers la recherche axée sur le patient et l’amélioration des accommodements pour les patients partenaires.

**Limites:**

Cette analyse ne relate que les points de vue de personnes faisant déjà partie du réseau. Bien que des personnes issues de la diversité fassent partie de notre réseau, certains groupes démographiques et leurs perspectives respectives pourraient être absents.

**Conclusion:**

Bon nombre des forces de Can-SOLVE CKD en matière d’EDIA reposent sur une culture établie d’inclusion des patients partenaires, sur le leadership intégré au réseau des peuples autochtones et des patients partenaires, ainsi que sur des structures de formation, d’intégration et de compensation pour les patients partenaires. Bien que les stratégies actuelles aient permis d’établir des bases solides, les prochaines étapes devront garantir une inclusion couvrant l’ensemble des groupes démographiques et des disciplines. Les suggestions des participants, telles que la mobilisation communautaire, un leadership diversifié et des communications multilingues, reflètent une volonté du réseau de mettre en œuvre des mesures concrètes pour renforcer les principes d’EDIA.

## Introduction

The consideration of equity, diversity, inclusion, and accessibility (EDIA), also commonly referred to as EDI, DEI, or IDEA, is critical for successfully improving kidney health research. In Canada, systemic inequities continue to contribute to significant disparities in access to dialysis,^1,2^ transplantation,^3,4^ and other health outcomes.^5-7^ Indigenous peoples are 66% less likely to receive a kidney transplant than Caucasian Canadians, despite a threefold higher risk of kidney failure.^3,4^ Similarly, African, Caribbean, and Black (ACB) and East Asian Canadians are 69% and 73% less likely to receive a living donor kidney transplant, respectively. These disparities are driven largely by health inequities due to geographic isolation, socioeconomic status, and systemic racism.

Patient-oriented research, which involves meaningful engagement of people with lived experience as co-investigators on research teams, offers a promising framework to advance EDIA.^8^ Since its inception in 2016, Canadians Seeking Solutions and Innovations to Overcome Chronic Kidney Disease (Can-SOLVE CKD) has meaningfully engaged people with lived experience into every aspect of the network,^9^ successfully contributing to inclusive and relevant research outputs.^10^

As part of the networks strategy to continuously improve through cycles of evaluation, reflection, and action-related amendments, an interactive network-wide EDIA audit was conducted from July 2023 to January 2024. This audit explored existing EDIA strengths and future directions through engagement with network members to collaboratively shape an EDIA strategy grounded in co-learning, transparency, and collective capacity-building.

## Methods

Early in 2023, a working group was established with eight members of the Can-SOLVE CKD network and led by an external EDIA expert (KTM), to conduct a network-wide audit to assess the integration and application of EDIA principles within the network’s culture and operations. Members of the Can-SOLVE CKD network, including patient partners, research team members, and staff, were invited to participate in one or more of seven interactive workshops held between June 2023 and January 2024 virtually on Zoom. Invitations were distributed via email to all members of the Can-SOLVE CKD network. Each workshop lasted 60 to 90 minutes. Prior to attending a workshop, participants received preparatory material in the form of an information booklet outlining the project objectives, workshop structure, definitions of EDIA, and a confidentiality statement (see Appendix 1).

At the beginning of each session, the facilitator (KTM) provided a verbal and visual review of the preparatory material and invited participants to complete an optional anonymous demographic poll to gather basic demographic information (participant role within the network, age category, gender identity, sexual orientation, race/ethnicity, and disability status). The facilitator guided the discussion sequentially through four domains of EDIA (Equity, Diversity, Inclusion, and Accessibility), exploring the network’s existing strengths and opportunities to improve within each area. Participants could contribute in one or more of the following ways^1^: anonymously posting comments using the Mural platform (LUMA Institute, Tactivos, Inc., 2024),^2^ typing responses in the Zoom chat, and^3^ speaking aloud using their microphone. All participants could view the live Mural board on their screen which was continuously updated by the facilitator and note-taker with participants’ comments in real time. Participants’ comments were represented as a virtual ‘sticky note’ on the live Mural board. Repeated or agreed-upon comments were displayed as multiple identical entries. A dedicated note-taker was present during each session to record supplement observations.

Individual participant consent was implied through participation in the workshops, but need for written informed consent was not required as workshops were conducted for internal network auditing purposes and no participant-sensitive or -identifying information were collected. All network members participating in this audit were informed that their feedback would be shared in aggregate form more broadly prior to participating in the workshops.

### Data Analysis

All digital sticky notes were exported into Microsoft Excel (Version 16.97.2, 2025 Microsoft) for data management and organization, including storage, review, coding, and search functions. A thematic analysis was conducted using an inductive approach to identify and interpret patterns within the data.^11^ Although input was collected under each EDIA domain, the analysis did not aim to isolate findings by domain. Rather, this structure was used to provide participants with multiple lenses through which to view recurring challenges or successes. A completed checklist of the Standards for Reporting Qualitative Research (SRQR) is provided in Appendix 2.

To ensure analytical rigor and reflect the depth of the dataset, data analysis was conducted collaboratively by five individuals representing diverse perspectives: two researchers (KTM and MDT), two patient partners, and two staff members (SA and MH). Each individual reviewed the digital sticky notes and generated preliminary codes. The coding process incorporated structured training and calibration to support team members with differing levels of qualitative research experience. This included an initial one-on-one meeting with the lead investigator (KTM) to review the coding framework and procedures, a follow-up one-on-one meeting after preliminary coding was completed to discuss reflections, and a final presentation by each coder to the working group. During this presentation, the working group provided feedback on coded items and engaged in discussion regarding the coders’ overarching interpretation of the dataset, which further informed the grouping of codes into themes. From these data, KTM and MDT independently developed a set of preliminary themes, which were then discussed and refined collaboratively. Final themes were reviewed in relation to both the coded data extracts and the dataset as a whole to ensure coherence and relevance.

## Results

### Participant Demographics

A total of seven workshops were conducted involving 49 participants: 16 patient partners, 19 research team members, and 14 staff. Across all workshops, 441 digital sticky notes were recorded and included in the analysis: 165 from patient partners, 144 from research team members, and 122 from staff. Of the 49 participants, 42 (86%) completed the optional demographics poll. The majority of respondents identified as women (79%), were under the age of 65 (71%), and identified as white (47%). A detailed table of participant demographics is provided in Appendix 3.

### Thematic Analysis

Two overarching categories were assessed through thematic analysis^1^: existing EDIA strengths and^2^ future directions to support EDIA. Within each category, three recurring themes emerged. For existing EDIA strengths, the following were identified^1^: inclusion and promotion of Indigenous voices and ways of knowing,^2^ patient partner participation, empowerment and leadership, and^3^ network-wide acceptance of EDIA principles. For future directions to support EDIA, the following recurring themes were identified^1^: increase diversity in perspectives and outreach methods,^2^ address biases in patient-oriented research, and^3^ refine accommodations. See Table 1 for participant comments organized by theme.

**Table 1.** Participant Comments Organized by Theme

Existing EDIA strengths
**Theme 1: Inclusion and promotion of Indigenous voices and ways of knowing**
“Incorporating and addressing Indigenous needs (i.e., land acknowledgement, diverse attendance at meetings)” – Patient partner
“IPERC national Indigenous representation…” – Research team member
“Indigenous led (etc Elders with lived experience)” – Research team member
“Presence of a home base” – Research team member
**Theme 2: Patient partner participation, empowerment, and leadership**
“Patient partner representation on leadership team” – Patient partner
“Patient-partners have the opportunity to give direct impact through stories” – Patient partner
“Lived experience as central to research and the main goal” – Research team member
“Patient voice and lived experience in all phases of the research… ensures the research we do meets the needs of clinical populations… adds richness and direction” – Research team member
**Theme 3: Network-wide acceptance of EDIA principles**
“Anti-racism programs, prayers and ceremonies to support work” – Patient partner
“Promotion of self-reflection… know expectations and how to relate to differences in culture” - Staff
“We are encouraged to share all voices in safe and inclusive ways… this allows for patient partners to have their say… no topic is off the table and I don’t think anyone feels like they ask “irrelevant” questions. All thoughts and comments are encouraged in a very supportive way” - Staff
“Safe space for chronically ill and disabled team members to ask for accommodations and no need to pretend you aren’t sick or disabled” - Staff

Future directions to support EDIA
**Theme 1: Increase diversity in perspectives and outreach methods**
“Rural involvement? Remote communities? How to get them at the table? Big challenge for inclusivity… looking for opportunities to have the patient voice” – Research team member
“Who is not present? Who should be present? How do you intentionally seek them out and explain why we need them there without it being tokenistic?” – Research team member
“Providing opportunities for underrepresented communities via scholarships, internships” – Patient partner
“Training regarding engagement with Black and South Asian populations. What organizations can we connect and learn from/with?” - Staff
**Theme 2: Recognizing capacity and autonomy in patient involvement**
“Categorization of a patient partner is limiting” – Patient partner
“I feel that we include a wide range of patient partners but we call on the same people and perhaps we need to include more patient partners to share the workload” – Research team member
“Barriers in understanding the network structure and resources for new team members to get a good idea of where to get involved within the network” – Research team member
“Heavy workload… hard to add onto that” – Patient partner
**Theme 3: Refine accommodations**
“Barriers for current workers… timing issues. Patient partners have skew toward senior patients who have more time available” – Patient partner
“Missing population due to timing issues… adjusting flexibility to incorporating different demands in life” – Patient partner
“Offer flexibility in rotating meetings… not as much stability in meeting times but gives you opportunity to choose which meetings you can participate in… participate but not at every meeting” – Research team member
“Patients have other commitments. Are we accessible to their time? Challenging to fit this in between their health appointments, waiting lists, and trying to make things happen. Very strong commitment but this is time intensive” - Research team member

### Existing EDIA Strengths

#### Theme 1: Inclusion and Promotion of Indigenous Voices and Ways of Knowing

Participants highlighted the exemplary work of the network in recognizing and addressing the unique cultures, needs, and colonial histories affecting First Nations, Métis, and Inuit people. The network took active measures to ensure that Indigenous voices are amplified within the network. Notably, the Indigenous Peoples’ Engagement and Research Council (IPERC), which includes national representation from various Indigenous communities, was described as integral to this success of the network. IPERC engages in traditional ceremonies, sharing circles, and other culturally relevant practices, fostering a deeper sense of connection and respect. Participants appreciated the network’s commitment to land acknowledgements and the incorporation of Indigenous Elders and Knowledge Keepers in meetings to ensure Indigenous voices are integrated into all levels of decision-making. The presence of a dedicated Indigenous-specific council and initiatives team, as well as the use of Indigenous-developed resources, were also noted as key contributors to creating a culturally supportive and culturally safe environment. Furthermore, the network-wide investment in cultural competency learning and training opportunities has enhanced the collective understanding, disrupted systemic racism and promoted respect for diverse cultural practices.

#### Theme 2: Patient Partner Participation, Empowerment, and Leadership

Participants unanimously agree that patient-partner perspectives are at the forefront of the network. Patient partners report that they are afforded opportunities to build new skills and to participate in numerous committees, including Can-SOLVE CKD Leadership and Steering Committees. Representation of patient partners at all levels, including leadership positions, was viewed as a notable strength of the network. The patient partner voice was described as central to research processes, with personal narratives foundational in guiding and shaping the work of the network. Patient partners also noted a meaningful integration of their lived experience and perspectives. Particularly, the Patient Governance Council, whose mandate is to ensure the patient voice is guiding the research work, was referred to as the “heart of the network”. Participants highlighted a strong support system provided by the Can-SOLVE CKD Operations Team for patient partners, including onboarding packages, training workshops, financial compensation and honoraria to attend and present at scientific conferences and meetings, and mentorship programs.

#### Theme 3: Network-wide Acceptance of EDIA Principles

Participants praised the network-wide commitment to EDIA principles, which are integrated into its structure and operations. At all levels, participants report the prioritization of anti-racism training, cultural safety, and respect for cultural practices. Participants noted the availability of training, workshops, and resources to promote self-reflection and understanding, efforts that fostered a supportive environment in which individuals felt safe to ask questions, share their voices, and engage in open dialogue. The intentional creation of a welcoming and inclusive workplace led to equitable team dynamics, characterized by a lack of hierarchy and an emphasis on collaboration. The network’s smaller Operations Team size was recognized as a key strength, enabling closer connections, communications, and a shared vision that valued inclusivity and mutual respect. Participants highlighted the safe work culture that provided opportunities for teams and committees to form deep connections, including connecting via in-person meetings and through non-work-related social events. Lastly, participants report the availability of explicit accommodations related to health-related disruptions, personal leave time, travel, and varied options for types of and levels of participation.

### Future Directions to Support EDIA

#### Theme 1: Increase Diversity in Perspectives and Outreach Methods

Participants unanimously expressed a strong desire to expand the diversity of perspectives and lived experience represented within the network, emphasizing the importance of including voices from underrepresented communities. The need was highlighted for increased representation of Indigenous and ACB nephrologists, as well as outreach to underserved populations, such as ACB communities, Asian committees, LGBTQ+ individuals, young mothers, and pediatric populations. Participants also called to continue the hiring of Indigenous and ACB managers to ensure leadership roles reflect the diversity of the communities served.

To achieve diverse representation, participants suggested several strategies; including direct community outreach efforts, such as forming partnerships with grassroots organizations and external networks already engaged with the identified underserved populations. Alternative recruitment strategies were proposed such as leveraging community leaders or hosting information sessions in local settings. Participants also emphasized the need for more accessible communication methods to engage diverse communities. Suggestions included improving access to translation services for multiple languages beyond French and English. Alternative avenues for communication, such as relevant messaging and collaborating with networks already trusted by these communities, were also recommended. Participants noted that intentional and sustained engagement is key to building trust and fostering authentic relationships with underserved groups.

#### Theme 2: Recognizing Capacity and Autonomy in Patient Involvement

Patient partners emphasized the importance of autonomy in defining their roles and level of involvement. They advocated against the assumption (placed upon them by the research team members and staff) that they face “workload burden” or “burnout”. Many patient partners expressed that they were and are capable of managing their boundaries and choosing how much they want to contribute. Participants also highlighted challenges related to a fragmented understanding of network opportunities. Limited awareness of available roles, committees, and initiatives was seen as a significant barrier to full engagement. Patient partners noted that they cannot meaningfully participate if they are unaware of opportunities that may be available to them. Clear communication about network opportunities and standardized comprehensive onboarding was identified as essential to ensuring equitable participation and allowing patient partners to be meaningfully engaged. Overall, workload concerns and a fragmented understanding of network opportunities contributed to suboptimal participation from newer patient partners.

#### Theme 3: Refine Accommodations

Participants highlighted the need for balanced accommodations to address time zones and varying schedules. Scheduling across time zones in a pan-Canadian network is particularly difficult, especially for patient partner volunteers who have day jobs and other health-related and caregiver demands on their time. Participants widely agreed that readjusting recurring meetings to varied weekdays and times can allow for maximum participation. While virtual opportunities were appreciated for accessibility, there was strong support for in-person meetings, valued for fostering deeper connections, enhancing communication, and improving collaboration. Hybrid formats, combining in-person gatherings with virtual streaming options, for Annual Meetings were proposed as an inclusive solution. Participants also emphasized the importance of accessibility accommodations, such as providing ramps and railings at venues, offering live content in multiple languages, and supporting those with visual and hearing impairments.

## Discussion

This network-wide EDIA audit is an important reflection on both the current successes and the areas requiring further attention within Can-SOLVE CKD.

A key strength identified was the inclusion and promotion of Indigenous voices and ways of knowing, largely driven by the impactful role of IPERC. The sustained commitment to cultural safety, ceremony, and representation is a model for how research networks can prioritize reconciliation and build authentic partnerships with Indigenous communities.^12-17^ This aligns with national calls to action in health research, including the Truth and Reconciliation Commission of Canada Calls to Action^18^ and Canadian Institutes of Health Research Institute (CIHR) of Indigenous Peoples’ Health Strategic Plan.^19^ The prominence of patient partners in leadership roles, supported by intentional onboarding, training, and compensation structures, reflects the success of embedding patient-oriented research principles throughout the network.^20^ These examples serve as tangible indicators of the network’s capacity to lead in EDIA-informed health research.

Despite these strengths, participants consistently identified the need to broaden representation, particularly among ACB, Asian, LGBTQ+, and pediatric communities. This must be a future priority. While current initiatives have fostered a strong cultural foundation,^10,20,21^ the next step should ensure that inclusivity extends across all demographic and disciplinary boundaries. It is also important to acknowledge that this audit is limited by the demographic variables used to characterize workshop attendees. Additional markers of diversity, such as level of education, socioeconomic indicators, and geographic context (e.g., rural or remote versus urban residence), were not captured, thereby limiting the comprehensive characterization of network members. Efforts focused on grassroots outreach, hiring diverse leadership, and improving multilingual communication, will demonstrate a readiness within the network to take actionable steps to improve EDIA. These approaches mirror best practices in equity-driven community engagement and align with Equity, Diversity, and Inclusion Action Plan of the CIHR.^22^

Another critical insight from the audit was the need to challenge biases in patient-oriented research, especially around assumptions about patient capacity, burnout, and workload. Patient partners emphasized their desire for autonomy and the right to determine their own level of engagement. This challenges the paternalistic tendency that is part of the current research culture and calls for a shift toward more reflexive, flexible, and transparent relationship-building across all roles.^23^ Additionally, the finding that patient partners require clear communication of opportunities and roles highlights the importance of knowledge translation and ensuring accessible language is a priority.

Finally, the feedback related to refining accommodations, especially regarding scheduling, virtual access, and accessibility, points to the complex nature of building and growing EDIA in research practice. Participants preferred hybrid models and adoption of flexibility for recurrent meeting times suggests a path forward to ensure maximum participation. The emphasis on physical accessibility and language translation also reinforces the need for EDIA strategies that account for both visible and invisible barriers to participation.

In summary, this audit demonstrates the value of a network-wide reflection on EDIA. It confirms that while strong foundations exist, continued efforts are required to refine a more diverse, equitable, and accessible research environment. By integrating the voices of all network members in this process, the network can move from reflection to action, ensuring that EDIA principles are not only embedded in structure but also continuously evolving through lived experience and collective insight.

## Supplemental Material

Supplemental Material - Advancing Equity, Diversity, Inclusion, and Accessibility in a Patient-Oriented Kidney Research Network: A Can-SOLVE CKD Program Report Supplemental Material for Advancing Equity, Diversity, Inclusion, and Accessibility in a Patient-Oriented Kidney Research Network: A Can-SOLVE CKD Program Report by Keila Turino Miranda, Jocelyn Jones, Melanie D. Talson, James Scholey, Julie Wysocki, Meghan J Elliott, Letitia Pokiak, Maria Torrejon, Heather Harris and Selina Allu in Canadian Journal of Kidney Health and Disease.

Supplemental Material - Advancing Equity, Diversity, Inclusion, and Accessibility in a Patient-Oriented Kidney Research Network: A Can-SOLVE CKD Program Report Supplemental Material for Advancing Equity, Diversity, Inclusion, and Accessibility in a Patient-Oriented Kidney Research Network: A Can-SOLVE CKD Program Report by Keila Turino Miranda, Jocelyn Jones, Melanie D. Talson, James Scholey, Julie Wysocki, Meghan J Elliott, Letitia Pokiak, Maria Torrejon, Heather Harris and Selina Allu in Canadian Journal of Kidney Health and Disease.

Supplemental Material - Advancing Equity, Diversity, Inclusion, and Accessibility in a Patient-Oriented Kidney Research Network: A Can-SOLVE CKD Program Report Supplemental Material for Advancing Equity, Diversity, Inclusion, and Accessibility in a Patient-Oriented Kidney Research Network: A Can-SOLVE CKD Program Report by Keila Turino Miranda, Jocelyn Jones, Melanie D. Talson, James Scholey, Julie Wysocki, Meghan J Elliott, Letitia Pokiak, Maria Torrejon, Heather Harris and Selina Allu in Canadian Journal of Kidney Health and Disease.
