# Supplementary material for: Advancing Equity, Diversity, Inclusion, and Accessibility in a Patient-Oriented Kidney Research Network: A Can-SOLVE CKD Program Report
Source: Can J Kidney Health Dis. 2026 May 25;13:20543581261455663. doi: 10.1177/20543581261455663 (PMC13201923; doi:10.1177/20543581261455663)
Supplement: Supplemental Material - Advancing Equity, Diversity, Inclusion, and Accessibility in a Patient-Oriented Kidney Research Network: A Can-SOLVE CKD Program Report [file sj-pdf-1-cjk-10.1177_20543581261455663.pdf]

Appendix 1. Workshop preparatory material information booklet.

Can-SOLVE CKD Network

IDEA WORKSHOP

CONFIDENTIALITY STATEMENT

During this workshop, **we prioritize the confidentiality and privacy of all participants**. To ensure a safe and open environment for discussion, please note the following:

- **Recording and Transcription**: The workshop sessions will not be recorded or transcribed in any form. We ask all participants to respect this policy and refrain from recording or transcribing the sessions themselves.

- **Anonymity and Feedback**: Any feedback provided during the workshop will be treated confidentially. ***We will not link feedback or comments to specific individuals within the network***. Your opinions and contributions will be considered in aggregate, with the aim of preserving anonymity and encouraging open dialogue.

By participating in this workshop, you agree to abide by these confidentiality guidelines. We appreciate your cooperation in maintaining a secure and respectful environment for all attendees.

PROJECT OVERVIEW

Goal: To diversify Can-SOLVE CKD's research environment to address inclusion, diversity, equity, and accessibility (IDEA) principles where perspectives of under-represented communities will be included.

Interactive Workshop
Develop an understanding of CanSOLVE CKD Phase 2 IDEA strengths and future directions.

Qualitative Analysis
To develop a mobilization plan to address IDEA principles and future directions within CanSOLVE CKD.

WORKSHOP STRUCTURE

Introduction

Overview of IDEA concepts &
Demographics Poll

Brainstorm

Participants will brainstorm main strengths and future directions within each branch of IDEA.

INTRODUCTION

Introduction	Overview of IDEA concepts & Demographics Poll

INCLUSION

- Active, intentional and continuous
- Addresses inequities in power & privilege
- Ensures welcoming spaces and opportunities for all
- Feeling of value and belongingness to organization

Example:

- "I am involved in decisions that impact my work at CanSOLVE"
- Tokenism

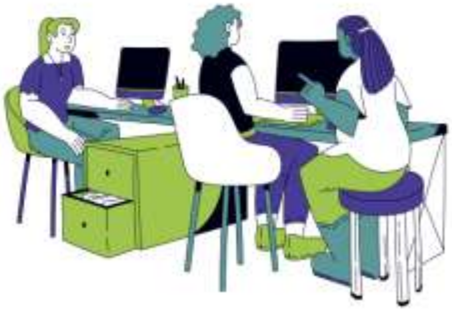

Can-SOLVE CKD Network

INTRODUCTION

Introduction — Overview of IDEA concepts & Demographics Poll

DIVERSITY

- Multiple perspectives, knowledge, and capabilities
- Quality in idea generation, innovation, problem-solving, and decision making
- Example:
- Do you believe there is a breadth of perspectives?
- What are some missing perspectives?
- What are some celebrated perspectives?

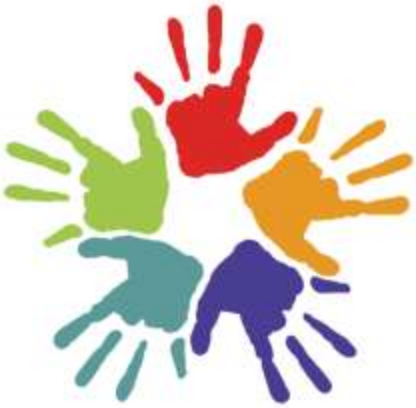

INTRODUCTION

Introduction

Overview of IDEA concepts & Demographics Poll

EQUITY

- Perception of fairness in skills, time, cognitive & emotional investment when compared to peers
- Proportional participation at all levels of CanSOLVE
- Accomodation to ensure individuals are provided with the tools necessary to thrive, which may differ from the needs of others
- Example:
- "I feel supported in my work compared to my peers"
- "My supervisor(s) treat(s) me the same as my peers"

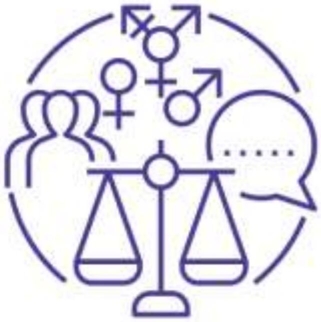

INTRODUCTION

Introduction

Overview of IDEA concepts & Demographics Poll

ACCESSIBILITY

- The ability to "access"
- Explicit and consistent accomodations
- Without accessibility, there is no opportunity to achieve equity, diversity or inclusion
- Example:
- Are virtual meetings accessible?
- Is contributing to CanSOLVE accessible?

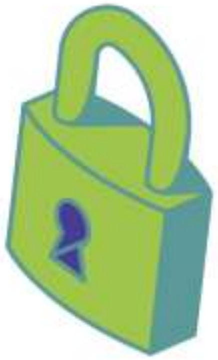

INTRODUCTION

Introduction	Overview of IDEA concepts & Demographics Poll

DEMOGRAPHICS POLL

As part of our commitment to inclusivity and understanding the diversity of voices in our workshop sessions, we will be conducting a demographics poll. The purpose of this poll is to gain insights into the representation of different groups within our community and identify any underrepresented voices.

- Please be assured that **we will not correlate individual answers with any demographic information.** All responses will be aggregated and anonymized to ensure the privacy and anonymity of all participants.

- Your cooperation in completing the demographics poll will greatly assist us in our efforts to create an inclusive and diverse workshop environment. Thank you for your understanding and participation.

Note: If you have any concerns regarding the demographics poll or the handling of your personal information, please reach out to [redacted] for further clarification or to discuss your preferences.

BRAINSTORM

Brainstorm

Participants will brainstorm main strengths and future directions within each branch of IDEA.

OUTCOMES

- Identify top 3 main strengths in CanSOLVE's IDEA initiatives.
- Identify top 3 future strategies to address IDEA principles.

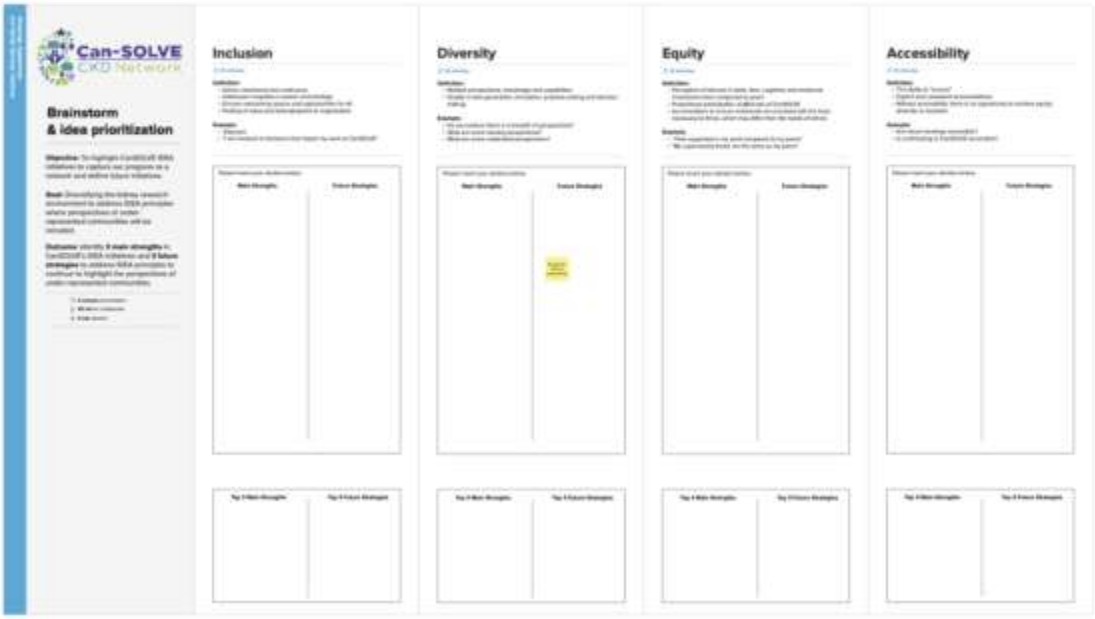

BRAINSTORM

Brainstorm

Participants will brainstorm main strengths and future directions within each branch of IDEA.

HOW TO PARTICIPATE

- Online Mural Board (link in chat)
  - Anonymous
- Zoom Chat (public or private)
- Unmute Microphone

HOW TO USE MURAL

- Enter the Mural Board prepared for the session (link will be provided in the chat)
  - Select Navigation settings: Trackpad Mode or Mouse Mode
- For each of the "sections" (IDEA) we will discuss the main strengths and future directions.
  - We will then "vote" on the top 3 main strengths and future directions (in Session 2).
- At the end, we expect having a list of tailored implementation strengths and future directions that can be considered.

IMPORTANT CONSIDERATIONS

We want YOUR perspective!

We want...

Your perspective as a patient-partner / clinician/researcher / staff

NOT

Your perspective taking into consideration ALL patient-partners / clinician/researchers / staff

IDEA WORKSHOP

For More Information: [redacted]
