# Supplementary material for: Advancing Equity, Diversity, Inclusion, and Accessibility in a Patient-Oriented Kidney Research Network: A Can-SOLVE CKD Program Report
Source: Can J Kidney Health Dis. 2026 May 25;13:20543581261455663. doi: 10.1177/20543581261455663 (PMC13201923; doi:10.1177/20543581261455663)
Supplement: Supplemental Material - Advancing Equity, Diversity, Inclusion, and Accessibility in a Patient-Oriented Kidney Research Network: A Can-SOLVE CKD Program Report [file sj-pdf-3-cjk-10.1177_20543581261455663.pdf]

Appendix 3. Participant demographics

Characteristic	Number
**Role in network**	
Patient partner	16
Research team member	19
Staff	14
**Age (years)**	
18-24	3
25-34	5
35-44	8
45-54	13
55-64	6
≥65	7
**Gender**	
Woman	33
Man	8
Prefer not to answer	1
**Gender identity**	
Cisgender	40
Two-Spirit	1
Prefer not to answer	1
**Sexual orientation**	
Heterosexual	34
Gay	1
Queer	1
Bisexual	1
Another sexual orientation	1
Prefer not to answer	4
**Identifies as an Indigenous person of North America**	
Yes	9
No	31
Prefer not to answer	2
**Race/ethnicity**	
White	23
South Asian/East Indian	3
Southeast Asian	2
Chinese, Filipino	1
Black	1
Other	7
Prefer not to answer	5
**Identifies as a person with disability**	
Yes, ongoing medical condition/chronic illness	8
Yes, physical disability or impairment	7
No	26
Prefer not to answer	1

Note. Demographic data available for 42 of 49 participants.
